# Atrial Fibrillation and Other Cardiovascular Factors and the Risk of Dementia: An Italian Case–Control Study

**DOI:** 10.3390/ijerph21060688

**Published:** 2024-05-27

**Authors:** Riccardo Mazzoli, Annalisa Chiari, Marco Vitolo, Caterina Garuti, Giorgia Adani, Giulia Vinceti, Giovanna Zamboni, Manuela Tondelli, Chiara Galli, Manuela Costa, Simone Salemme, Giuseppe Boriani, Marco Vinceti, Tommaso Filippini

**Affiliations:** 1 Environmental, Genetic and Nutritional Epidemiology Research Center (CREAGEN), Department of Biomedical, Metabolic and Neural Sciences, University of Modena and Reggio Emilia, 41225 Modena, Italy; riccardo.mazzoli@unimore.it (R.M.);; 2 Neurology Unit, Baggiovara Hospital, AOU Modena, 41126 Modena, Italy; 3 Cardiology Division, Department of Biomedical, Metabolic and Neural Sciences, University of Modena and Reggio Emilia, Policlinico di Modena, 41124 Modena, Italy; 4 Post Graduate School of Pediatrics, Department of Medical and Surgical Sciences for Mothers, Children and Adults, University of Modena and Reggio Emilia, 41124 Modena, Italy; 5 Department of Biomedical, Metabolic, and Neural Sciences, University of Modena and Reggio Emilia, 41125 Modena, Italy; 6 Primary Care Department, Modena Local Health Authority, 41124 Modena, Italy; 7 Neurology Unit of Carpi Hospital, Modena Local Health Authority, 41012 Carpi, Italy; 8 School of Advanced Studies, University of Camerino, 62032 Camerino, Italy; 9 Department of Epidemiology, Boston University School of Public Health, Boston, MA 02118, USA; 10 School of Public Health, University of California Berkeley, Berkeley, CA 94704, USA

**Keywords:** atrial fibrillation, cardiovascular factors, case–control study, dementia, diabetes, dyslipidemia, early-onset dementia, late-onset dementia

## Abstract

Dementia is a major neurologic syndrome characterized by severe cognitive decline, and it has a detrimental impact on overall physical health, leading to conditions such as frailty, changes in gait, and fall risk. Depending on whether symptoms occur before or after the age of 65, it can be classified as early-onset (EOD) or late-onset (LOD) dementia. The present study is aimed at investigating the role of cardiovascular factors on EOD and LOD risk in an Italian population. Using a case–control study design, EOD and LOD cases were recruited at the Modena Cognitive Neurology Centers in 2016–2019. Controls were recruited among caregivers of all the dementia cases. Information about their demographics, lifestyles, and medical history were collected through a tailored questionnaire. We used the odds ratio (OR) and 95% confidence interval (CI) to estimate the EOD and LOD risk associated with the investigated factors after adjusting for potential confounders. Of the final 146 participants, 58 were diagnosed with EOD, 34 with LOD, and 54 were controls. According to their medical history, atrial fibrillation was associated with increased disease risk (ORs 1.90; 95% CI 0.32–11.28, and 3.64; 95% CI 0.32–41.39 for EOD and LOD, respectively). Dyslipidemia and diabetes showed a positive association with EOD, while the association was negative for LOD. We could not evaluate the association between myocardial infarction and EOD, while increased risk was observed for LOD. No clear association emerged for carotid artery stenosis or valvular heart disease. In this study, despite the limited number of exposed subjects and the high imprecision of the estimates, we found positive associations between cardiovascular disease, particularly dyslipidemia, diabetes, and atrial fibrillation, and EOD.

## 1. Introduction

Dementia is a neurological syndrome characterized by an acquired cognitive decline that is severe enough to interfere with daily functioning [1,2], leading to increased fall risk, changes in gait, and overall physical deterioration, which further complicate the patient’s care and quality of life.

There are several causes of dementia, which can be classified according to the specific underlying pathology. Dementia encompasses a heterogeneous group of diseases, with Alzheimer’s disease (AD), vascular dementia, dementia with Lewy bodies, and frontotemporal dementia (FTD) being the most common underlying pathologies [3]. Regarding EOD, the most common diagnosis is Alzheimer’s dementia (AD), followed by frontotemporal dementia (FTD) and vascular dementia [4,5]. Other causes, such as progressive supranuclear palsy, Lewy’s body dementia, or Parkinson’s disease with dementia, are less common.

Additionally, dementia can be classified based on the age of symptom onset: early-onset dementia (EOD) is defined when symptoms occur before the age of 65, while symptom occurrence after the age of 65 indicates late-onset dementia (LOD) [6,7]. With societal aging, the prevalence of dementia is expected to rise, with some estimates projecting a two-fold increase in cases over the next decade [8]. The impact of dementia is significant, affecting both patients and their families, with caregivers experiencing a substantial burden [9]. For patients with EOD, the toll is often greater; given that they continue to bear substantial economic and social responsibilities [10], their employment and income are often more severely affected [11,12], and they more frequently have young children as it is also increasingly common to become a parent later in life [13].

The genetic influence in dementia is well recognized. Familial AD has been associated with autosomal dominant mutations of *APP*, *PSEN1*, and *PSEN2*, while more than 550 genes have been implicated in AD risk modulation [14], with the *APOE* ε4 allele being a major genetic risk factor [15,16,17]. FTD has a genetic cause in 30–40% of cases, with *MAPT* and *PGRN* mutations and *C9orf72* expansion being the more frequent [18]. Despite genetic susceptibility playing a role in dementia’s etiology, known gene mutations explain only fewer than 10% of dementia cases. As a result, additional factors, including environmental and occupational exposures, as well as lifestyle and nutritional choices, may be implicated [19,20,21,22,23,24] and may even carry a greater weight in EOD than in LOD [25,26].

There is evidence that dementia is linked to a history of cardiovascular disease (CVD) [27], such as heart failure [28], coronary heart disease [29], and atrial fibrillation (AF) [30,31,32]. However, several studies have yielded inconclusive results, with also evidence of differences between EOD and LOD [33]. The interpretation of these data is challenging, especially given that cardiovascular risk factors, such as hypertension, are often underdiagnosed, and individuals monitored in hospitals for other medical conditions are more readily diagnosed with dementia. Italy serves as an interesting case study for observing these factors, as it has not only the highest proportion of elderly individuals in Europe [34], but it is also experiencing a rise in CVD prevalence across all age groups [35].

The aim of this study is to investigate the relation between CVDs and the risk of both EOD and LOD in order to explore possible differences due to the age of onset.

## 2. Methods

### 2.1. Study Population

This study has been approved by the Modena Hospital Ethics Committee (approval no. 186/2016). Using a case–control study design, we recruited recently diagnosed EOD and LOD cases resident in the Modena province who were referred to either of the two Cognitive Neurology Centers of the Modena province in Northern Italy—the Policlinico University Hospital and Carpi Hospital—from 2016–2019 [19,36]. These centers are tasked with managing subjects with cognitive impairment and dementia, and they represent the totality of the dementia care network in the Modena province, which is part of the nationwide network under the Italian National Health System. Referrals to these centers can be made by general practitioners or specialists. At these centers, patients are monitored periodically from the time of admission and/or diagnosis, with follow-up visits every 6–12 months. Diagnosis of dementia is carried out according to the criteria outlined in the Diagnostic and Statistical Manual of Mental Disorders, 5th Edition (DSM-5) [37], ensuring consistency in determining the specific clinical diagnosis (e.g., AD, FTD, vascular dementia, etc.) and the timing of symptom onset (EOD vs. LOD).

We included cases where dementia was the principal cause of disability, regardless of the dementia subtype, specifically Alzheimer’s dementia, dementia with Lewy bodies, and frontotemporal dementia [38,39], in order to recruit a comparable sample of subjects with dementia as the primary disease. Therefore, we excluded subjects with coexisting diagnoses of pervasive developmental disorders, major psychiatric disorders, or cognitive impairment in the context of another neurological disorders (e.g., multiple sclerosis or cerebrovascular disease with severe motor disability) [19]. We classified the cases based on whether the dementia was diagnosed with symptoms before the age of 65 (EOD) or after the age of 65 (LOD). We recruited the control population from the caregivers of dementia patients referred to the same centers and administered the same questionnaire to them.

### 2.2. Exposure Assessment

Each subject received a tailored questionnaire collecting anamnestic data and information about lifestyle factors, with special attention to factors possibly related to dementia [40]. The questionnaire was divided into six sections, each investigating a specific aspect of the patient’s life: (1) demographics; (2) detailed medical history; (3) occupational history; (4) non-occupational activities, such as hobbies or sports; (5) residential history. Additionally, female individuals completed a section featuring questions about previous pregnancies, menopausal status, and the use of hormone replacement therapy [19].

For medical history, we included specific questions to assess the occurrence of clinical conditions prior to the diagnosis of dementia, with a focus on cardiovascular risk factors and diseases, namely atrial fibrillation, carotid artery stenosis, diabetes, dyslipidemia, hypertension, myocardial infarction, stroke, and valvular heart disease.

Smoking exposure was thoroughly investigated by assessing if the subjects had smoked in the last six months and, if so, how old they were when they started smoking and how many cigarettes they smoked on average per day. If they did not smoke, we further asked how old they were when they quit smoking definitively. The responses were then used to classify the population into two categories, those who had smoked and those who had not, to facilitate the examination of smoking behavior as a potential confounder.

The assessment and validation of the medical history related to other clinical conditions were conducted by accessing medical hospital records and outpatient visits.

### 2.3. Statistical Analysis

We used multivariate unconditional logistic regression models to calculate the odds ratio (OR) and its corresponding 95% confidence interval (CI) for both EOD and LOD in relation to the investigated factors. We incorporated sex, age (in years), and education (years) into the multivariable model as potential confounders and effect modifiers. We also performed sensitivity analyses with the addition of smoking habits (never vs. ever) to the adjustment factors. Whenever possible, we conducted stratified analyses based on clinical dementia subtypes, specifically early-onset Alzheimer’s dementia (EO-AD), early-onset frontotemporal dementia spectrum (EO-FTD), and late-onset Alzheimer’s dementia (LO-AD). Finally, we also performed a sensitivity analysis with mutual adjustment for a history of stroke and AF when computing their risk estimates given their close clinical relation [41,42,43]. All analyses were performed using the statistical package Stata-18.0 (Stata Corp., College Station, TX, USA, 2023).

## 3. Results

The final study sample encompassed 146 participants, including 58 (male/female: 25/33) subjects with EOD, 34 (male/female: 15/19) subjects with LOD, and 54 (male/female: 23/31) controls.

Table 1 outlines the sociodemographic characteristics of the study participants, along with their education level, marital status, and smoking habits. The mean age at EOD onset was 59 years (standard deviation SD: 5.0), while the mean age at the onset of LOD was 74 years (SD: 4.0). Achieving a high-school education was noted in 32.8% of EOD cases, in 17.7% of LOD cases, and in 38.9% of controls. Conversely, 5.2%, 8.8%, and 20.4% of EOD, LOD, and control patients, respectively, had attained a university education or higher. The prevalence of individuals with a history of smoking was elevated among both EOD cases and controls. Specifically, 60.3% of EOD cases and 55.6% of controls had smoked, with EO-FTD having the highest percentage of ever-smokers at 63.2%. Conversely, LOD cases exhibit a greater proportion of non-smokers (55.9%).

Regarding dementia diagnoses, Alzheimer’s dementia was the most prevalent for both EOD and LOD cases (55.2% and 73.5%, respectively), followed by FTD (32.8% and 5.9%), although the latter was less represented among LOD cases (Supplementary Table S1), thus hampering further evaluation in the subsequent risk analysis.

History of several cardiovascular risk factors (Table 2) revealed a reduced risk for both EOD and LOD in the case of hypertension (ORs 0.54; 95% CI 0.23–1.24 and 0.55; 95% CI 0.11–2.70, respectively). In contrast, dyslipidemia and diabetes exhibited an elevated risk with EOD only (ORs 1.43; 95% CI 0.63–3.27 and 2.72; 95% CI 0.67–10.97, respectively). Conversely, a decreased risk was found with LOD (ORs 0.31, 95% CI 0.05–1.91 and 0.70; 95% CI 0.08–5.98). When assessing the history of major cardiovascular diseases, no association could be evaluated between myocardial infarction and EOD prevalence, as no cases reported a positive history. Conversely, we highlighted an increased risk for LOD (OR 2.43; 95% CI 0.16–36.26), with a substantial but imprecise risk increase in LOD-AD (OR 7.05; 95% CI 0.29–171.31). History of stroke was associated with increased risk of EOD (OR 3.78; 95% CI 0.39–36.76) but not with LOD. A positive association was observed between a pre-existing diagnosis of AF and both EOD (OR 1.90; 95% CI 0.32–11.28) and LOD (OR 3.64; 95% CI 0.32–41.39), with a similar risk increase in the AD and FTD subtypes. Finally, no clear association emerged between carotid artery stenosis and both EOD and LOD despite an indication of imprecisely decreased risk (OR 0.79; 95% CI 0.24–2.61 for EOD and OR 0.50; 95% CI 0.05–4.61 for LOD), nor was one detected for valvular heart disease, since only one control and one EO-AD case had a positive history.

The sensitivity analysis adjusting AF for stroke and vice versa is reported in Table 3, which substantially confirmed such an increase in risk. The risk of dementia associated with stroke, when adjusted for AF, revealed an association with EO and EO-AD (OR 4.01; 95% CI 0.41–39.19 and 3.81; 95% CI 0.29–50.23, respectively). Similarly, the risk associated with AF adjusted for stroke confirmed the increased risk already observed for both EOD and LOD (OR 2.07; 95% CI 0.35–12.27 and 3.43; 95% CI 0.31–38.33, respectively).

## 4. Discussion

In this case–control study in Modena, we investigated the relation between the history of cardiovascular disease and the risk of dementia, also based on its age of onset and main subtypes.

Dementia has a complex etiopathogenesis involving the interplay between genetic, neuropathologic, and environmental factors. Particularly in the context of EOD, genetic factors are acknowledged as robust contributors to disease risk. However, the prevalence of familial dementia is rather low, and only a minor fraction of EOD cases can be linked to familial factors [44]. Studies have indicated that cardiovascular risk factors are more relevant than potential genetic causes in EOD [45]. For instance, one study emphasized that strokes increase the risk of developing EOD by about threefold, while another study showed a high prevalence of stroke or transient ischemic attack (TIA) history in individuals with EOD compared with the general population. A higher dementia risk, regardless of age of onset, was found in individuals suffering from coronary heart disease, AF, and stroke [46]. Interestingly, a prospective cohort study conducted with over thirty years of follow-up has highlighted how the association between CVD and the onset of dementia appears to be particularly relevant among individuals who experience CVD events before the age of 60 [47].

In our study population, hypertension was associated with a decreased risk of dementia, both EOD and LOD. These findings conflict with previous results and especially with the recognized role of hypertension in the etiology of vascular dementia, being linked to cerebral small vessel disease, a primary cause of lacunar stroke and a noteworthy contributor to vascular-related cognitive impairment [48]. However, it must be noted that the evidence about hypertension in younger adults and in older adults aged ≥80 years is much more limited, with hypertension occurring later in life being linked to a lower risk of developing dementia [49,50]. Nonetheless, our different results were likely related to our selection criteria, which excluded cases with cognitive impairment within a coexisting condition causing motor deficits, such as stroke. The negative association may indicate that treatment for hypertension can be beneficial and can decrease the risk of other forms of dementia not related to stroke, consistent with the stratified analyses limited to AD and FTD indicating an even lower disease risk. Our finding could also be related to a higher detection of hypertension in subjects with dementia compared with controls, especially in its early phase. Due to the study design, the possibility of such detection bias cannot be entirely ruled out, as cardiovascular disease might be undetected or under-reported in control subjects. On the other hand, this is unlikely to occur for cardiovascular diseases such as stroke, and particularly for AF, which more frequently has a self-evident clinical presentation.

The results we found in our population confirmed the positive association between stroke and EOD, particularly in EO-AD. Despite the mutual influence of stroke and dementia in their pathophysiology and development not being clearly understood, they were demonstrated to share similar risk factors, including genetic predispositions, some comorbidities, and also similar histological alterations [51,52]. In addition, subjects with AD experience a higher incidence of strokes, more severe outcomes, and higher mortality compared with non-AD patients [53,54]. Stroke showed a significant role in dementia’s etiology irrespective of the concomitant presence of AF, particularly in the case of EOD [46]. Interestingly, also in our population, the positive association was confirmed after further adjusting for AF, although this was limited by the high imprecision of the estimates. This could be partially attributed to our study design, as patients with post-stroke dementia (i.e., who had strokes severe enough to permanently impair cognitive function in otherwise previously healthy individuals and with concomitant motor impairment) were purposely excluded. This approach limited the number of subjects reporting stroke in their medical history, as well as stratified the analysis for EO-FTD or LOD, and while this may have ensured a more homogeneous sample that allowed for comparisons between types of cognitive impairment not directly caused by a stroke episode, it may also have overlooked interactions between strokes and dementia, limiting the overall generalizability of our findings, as stroke and dementia often share a common pathogenic mechanism.

In our study, a history of diabetes and dyslipidemia revealed an increased risk of EOD for both the EO-AD and EO-FTD forms, particularly the latter. Conversely and unexpectedly, for LOD, there was a negative association with both diabetes and dyslipidemia, except for the weakly positive association between LOD-AD and dyslipidemia. This appears consistent with the notion that the factors influencing dementia development vary based on the age of onset: the younger the patient, the greater the probability that genetic or metabolic conditions could be at the root of the pathological condition [55]. This result, however, needs to be weighed against the evidence emerging from other studies showing a positive association between diabetes and late-onset dementia, and a higher risk of developing dementia when individuals were affected at a young age, regardless of its subtype. More specifically, research suggests that an earlier onset of diabetes is linked with a higher risk of dementia [56], and that the risk of AD is higher among people with diabetes than in the general population, irrespective of the age of onset [57]. Concerning diabetes, various studies have suggested that it has a role in increasing the risk of developing vascular dementia and AD, perhaps due to direct effects of the intrinsic metabolic conditions in diabetes (e.g., hyperglycemia and hyperinsulinemia) or through the mediating effect of other disorders [58]. For instance, it has been noted that the additional risk for vascular dementia, but not for nonvascular dementia, seems to be greater in women who also suffer from an increased AD and AD-related dementia mortality [59]. With regards to AF, the results of our study are consistent with other evidence suggesting its association with a higher risk of dementia. In particular, our findings suggest a positive association with both EOD and LOD, but they are stronger for the latter [60,61]. Additionally, age at AF onset appears to play a relevant role. Notably, some studies reported that an earlier AF onset was associated with a higher risk of subsequent all-cause dementia later in life [33] or found that the risk of dementia due to AF was greater in younger participants [61], while other studies reported such an association only for AF that first occurred in late-life [62]. Interestingly, a meta-analysis implementing a stratified analysis by age of onset of EOD reported a positive association between a pre-existing AF diagnosis and dementia risk in all age groups, although a negative association was noted for subjects aged ≤55 years [32]. Despite the mechanism linking a higher risk of developing EOD in individuals with AF not yet being fully clear, observational studies investigating the effects of AF therapy on dementia prevention have indicated a correlation between the use of oral anticoagulants and a reduced risk of both dementia and cognitive impairment in individuals with AF [63,64]. Various mechanisms have been suggested for the relation between AF and dementia, including changes in cerebral blood flow, rhythm-related brain hypoperfusion, embolic events, and oxidative injury [65].

This study has some strengths. Firstly, the study was conducted on a population with a definite diagnosis of dementia, with accurate methodologies in specialized centers for the evaluation and diagnosis of subjects with cognitive impairment, and it covers the entire population of the Modena province. Moreover, this is, to the best of our knowledge, the first study conducted in Italy on the role of cardiovascular risk factors in the etiology of EOD and LOD, and one of the very few carried out on the relation between CVD and specific dementia subtype. Regarding the study limitations, a concern pertains to the statistical imprecision of the estimates due to the limited number of study subjects. For these reasons, some of the study findings should be interpreted with caution due to their wide confidence intervals. However, considering the rarity of EOD, even in the case of a few exposed subjects, the results of this study provide valuable insights for future research. A larger sample size could enable stratification by age groups, which could be of interest for EOD in particular, given the varying age-specific prevalence of EOD subtypes [66]. A potential selection bias in the selection of the controls, who were recruited from among the caregivers of cases, must also be acknowledged. Regarding the cases, the deliberate exclusion of cognitive decline directly from stroke might have led to an underestimation of the actual risk, which must be noted. Finally, information bias in assessing the clinical history could also have occurred, calling for the use of validated clinical databases to allow for a more accurate evaluation of the history of cardiovascular disease and its associated treatments.

## 5. Conclusions

In this study, despite the limited number of exposed subjects and the high imprecision of the estimates, we found a positive association between CVD, particularly dyslipidemia, diabetes and atrial fibrillation, and EOD. Regarding atrial fibrillation, an even stronger association is noted with LOD, further confirming its role in the pathogenesis of later forms of dementia.

## Figures and Tables

**Table 1:** Sociodemographic characteristics of study subjects. Values are the number (N) and percentage (%), when not differently reported. EOD, early-onset dementia; EO-AD, early-onset Alzheimer’s dementia; EO-FTD, early-onset frontotemporal dementia; LOD, late-onset dementia; LO-AD, late-onset Alzheimer’s dementia.

Characteristics	Controls	EOD	EO-AD	EO-FTD	LOD	LO-AD
	N = 54	N = 58	N = 32	N = 19	N = 34	N = 25
Age at questionnaire filling						
Mean (SD)	63.8 (9.6)	65.6 (5.2)	65.8 (4.5)	65.8 (5.7)	80.8 (6.3)	81.4 (5.7)
<65 years	28 (51.9)	22 (37.9)	12 (37.5)	6 (31.6)	-	-
>65 years	26 (48.2)	36 (62.1)	20 (62.5)	13 (68.4)	34 (100)	25 (100)
Age at disease onset						
Mean (SD)	-	59.3 (4.7)	59.7 (4.1)	59.1 (5.1)	74.2 (4.4)	77.7 (4.8)
Sex						
Males	23 (42.6)	25 (43.1)	11 (34.4)	11 (57.9)	15 (44.1)	9 (36.0)
Females	31 (57.4)	33 (56.9)	21 (65.6)	8 (42.1)	19 (55.9)	16 (64.0)
Educational attainment						
Primary school or less	11 (20.4)	16 (27.6)	8 (25.0)	5 (26.3)	15 (44.1)	11 (44.0)
Middle school	11 (20.4)	20 (34.5)	10 (31.3)	5 (42.1)	10 (29.4)	7 (28.0)
High school	21 (38.9)	19 (32.8)	12 (37.5)	5 (26.3)	6 (17.7)	4 (16.0)
College or more	11 (20.4)	3 (5.2)	2 (6.3)	1 (5.3)	3 (8.8)	3 (12.0)
Marital status						
Married/unmarried partner	48 (88.9)	48 (82.8)	25 (78.1)	17 (89.5)	25 (73.5)	19 (76.0)
Single	3 (5.6)	1 (1.7)	1 (3.1)	-	-	-
Separated/divorced	2 (3.7)	3 (5.2)	-	2 (10.5)	-	-
Widowed	1 (1.8)	6 (10.3)	6 (18.8)	-	9 (26.5)	6 (24.0)
Smoking habits						
Ever	30 (55.6)	35 (60.3)	19 (59.4)	12 (63.2)	15 (44.1)	10 (40.0)
Never	24 (44.4)	23 (39.7)	13 (40.6)	7 (36.8)	19 (55.9)	15 (60.0)

**Table 2:** Risk of dementia for investigated cardiovascular risk factors and diseases. Odds ratio (OR) and 95% confidence interval (CI), adjusted for sex, age (in years), educational attainment (in years of education), and smoking habits (ever or never smoker). EOD, early-onset dementia; EO-AD, early-onset Alzheimer’s dementia; EO-FTD, early-onset frontotemporal dementia; LOD, late-onset dementia; LO-AD, late-onset Alzheimer’s dementia.

Disease	Cases (y/n)	Controls (y/n)	OR	(95% CI)
Atrial fibrillation				
EOD	5/53	2/52	1.90	0.32–11.28
EO-AD	3/29	2/52	2.15	0.31–15.02
EO-FTD	2/17	2/52	2.52	0.27–23.49
LOD	6/28	2/52	3.64	0.32–41.39
LO-AD	3/22	2/52	7.43	0.31–175.94
Carotid artery stenosis				
EOD	6/52	7/47	0.79	0.24–2.61
EO-AD	3/29	7/47	0.85	0.19–3.76
EO-FTD	2/17	7/47	0.82	0.15–4.56
LOD	2/32	7/47	0.50	0.05–4.61
LO-AD	1/24	7/47	0.33	0.02–6.60
Diabetes				
EOD	9/49	3/51	2.72	0.67–10.97
EO-AD	3/29	3/51	1.50	0.27–8.46
EO-FTD	3/16	3/51	2.32	0.39–13.90
LOD	3/31	3/51	0.70	0.08–5.98
LO-AD	2/23	3/51	0.64	0.04–9.83
Dyslipidemia				
EOD	22/36	17/37	1.43	0.63–3.27
EO-AD	11/21	17/37	1.18	0.44–3.15
EO-FTD	8/11	17/37	1.55	0.50–4.76
LOD	14/20	17/37	0.31	0.05–1.91
LO-AD	13/12	17/37	0.47	0.06–3.59
Hypertension				
EOD	19/39	22/32	0.54	0.23–1.24
EO-AD	9/23	22/32	0.37	0.13–1.05
EO-FTD	4/15	22/32	0.25	0.06–0.98
LOD	17/17	22/32	0.55	0.11–2.70
LO-AD	13/22	22/32	0.78	0.12–5.30
Myocardial infarction				
EOD	0/58	3/51	-	-
EO-AD	0/32	3/51	-	-
EO-FTD	0/19	3/51	-	-
LOD	3/31	3/51	2.43	0.16–36.26
LO-AD	3/22	3/51	7.05	0.29–171.31
Stroke				
EOD	4/54	1/53	3.78	0.39–36.76
EO-AD	2/30	1/53	3.50	0.27–45.91
EO-FTD	0/19	1/53	-	-
LOD	0/34	1/53	-	-
LO-AD	0/25	1/53	-	-
Valvular heart disease				
EOD	1/57	1/53	0.83	0.04–15.22
EO-AD	1/31	1/53	1.74	0.09–34.25
EO-FTD	0/19	1/53	-	-
LOD	0/34	1/53	-	-
LO-AD	0/25	1/53	-	-

**Table 3:** Risk of dementia for stroke and atrial fibrillation with mutual adjustment, which was further adjusted by sex, age (in years), educational attainment (in years of education), and smoking habits. Odds ratio (OR) and 95% confidence interval (CI). EOD, early-onset dementia; EO-AD, early-onset Alzheimer’s dementia; EO-FTD, early-onset frontotemporal dementia; LOD, late-onset dementia; LO-AD, late-onset Alzheimer’s dementia.

Disease	Cases (y/n)	Controls (y/n)	OR	(95% CI)
Stroke				
EOD	4/54	1/53	4.01	0.41–39.19
EO-AD	2/30	1/53	3.81	0.29–50.23
EO-FTD	0/19	1/53	-	-
LOD	0/34	1/53	-	-
LO-AD	0/25	1/53	-	-
Atrial Fibrillation				
EOD	5/53	2/52	2.07	0.35–12.27
EO-AD	3/29	2/52	2.33	0.33–16.35
EO-FTD	2/17	2/52	2.42	0.26–22.54
LOD	6/28	2/52	3.43	0.31–38.33
LO-AD	3/22	2/52	6.92	0.29–165.41

## Data Availability

The data presented in this study cannot be made available due to privacy restrictions regarding the personal and clinical data of participants.

## Supplementary Materials

The following supporting information can be downloaded at: https://www.mdpi.com/article/10.3390/ijerph21060688/s1, Table S1: Clinical diagnosis of early-onset (EOD) and late-onset (LOD) dementia cases.

## References

[1] Gale S.A., Acar D., Daffner K.R. (2018). Dementia. Am. J. Med..

[2] American Psychiatric Association (2013). Neurocognitive Disorders. Diagnostic and Statistical Manual of Mental Disorders.

[3] Cao Q., Tan C.C., Xu W., Hu H., Cao X.P., Dong Q., Tan L., Yu J.T. (2020). The Prevalence of Dementia: A Systematic Review and Meta-Analysis. J. Alzheimers Dis..

[4] Chiari A., Vinceti G., Adani G., Tondelli M., Galli C., Fiondella L., Costa M., Molinari M.A., Filippini T., Zamboni G. (2021). Epidemiology of early onset dementia and its clinical presentations in the province of Modena, Italy. Alzheimers Dement..

[5] Kvello-Alme M., Brathen G., White L.R., Sando S.B. (2019). The Prevalence and Subtypes of Young Onset Dementia in Central Norway: A Population-Based Study. J. Alzheimers Dis..

[6] Giannakopoulos P., Hof P.R., Savioz A., Guimon J., Antonarakis S.E., Bouras C. (1996). Early-onset dementias: Clinical, neuropathological and genetic characteristics. Acta Neuropathol..

[7] Guerreiro R., Gibbons E., Tabuas-Pereira M., Kun-Rodrigues C., Santo G.C., Bras J. (2020). Genetic architecture of common non-Alzheimer’s disease dementias. Neurobiol. Dis..

[8] GBD Collaborators (2021). Global mortality from dementia: Application of a new method and results from the Global Burden of Disease Study 2019. Alzheimers Dement.

[9] Cheng S.T. (2017). Dementia Caregiver Burden: A Research Update and Critical Analysis. Curr. Psychiatry Rep..

[10] Ducharme F., Kergoat M.J., Antoine P., Pasquier F., Coulombe R. (2013). The unique experience of spouses in early-onset dementia. Am. J. Alzheimers Dis. Other Demen.

[11] Sakata N., Okumura Y. (2017). Job Loss After Diagnosis of Early-Onset Dementia: A Matched Cohort Study. J. Alzheimers Dis..

[12] Chiari A., Pistoresi B., Galli C., Tondelli M., Vinceti G., Molinari M.A., Addabbo T., Zamboni G. (2021). Determinants of Caregiver Burden in Early-Onset Dementia. Dement. Geriatr. Cogn. Dis. Extra.

[13] Sikes P., Hall M. (2018). The impact of parental young onset dementia on children and young people’s educational careers. Br. Educ. Res. J..

[14] Neuner S.M., Tcw J., Goate A.M. (2020). Genetic architecture of Alzheimer’s disease. Neurobiol. Dis..

[15] Riedel B.C., Thompson P.M., Brinton R.D. (2016). Age, APOE and sex: Triad of risk of Alzheimer’s disease. J. Steroid Biochem. Mol. Biol..

[16] Robinson M., Lee B.Y., Hane F.T. (2017). Recent Progress in Alzheimer’s Disease Research, Part 2: Genetics and Epidemiology. J. Alzheimers Dis..

[17] Hendriks S., Ranson J.M., Peetoom K., Lourida I., Tai X.Y., de Vugt M., Llewellyn D.J., Kohler S. (2024). Risk Factors for Young-Onset Dementia in the UK Biobank. JAMA Neurol..

[18] Siuda J., Fujioka S., Wszolek Z.K. (2014). Parkinsonian syndrome in familial frontotemporal dementia. Park. Relat. Disord..

[19] Adani G., Filippini T., Garuti C., Malavolti M., Vinceti G., Zamboni G., Tondelli M., Galli C., Costa M., Vinceti M. (2020). Environmental risk factors for early-onset Alzheimer’s dementia and frontotemporal dementia: A case-control study in Northern Italy. Int. J. Environ. Res. Public Health.

[20] Fenoglio C., Scarpini E., Serpente M., Galimberti D. (2018). Role of genetics and epigenetics in the pathogenesis of Alzheimer’s disease and frontotemporal dementia. J. Alzheimers Dis..

[21] Bosi M., Malavolti M., Garuti C., Tondelli M., Marchesi C., Vinceti M., Filippini T. (2022). Environmental and lifestyle risk factors for early-onset dementia: A systematic review. Acta Biomed..

[22] Livingston G., Huntley J., Sommerlad A., Ames D., Ballard C., Banerjee S., Brayne C., Burns A., Cohen-Mansfield J., Cooper C. (2020). Dementia prevention, intervention, and care: 2020 report of the Lancet Commission. Lancet.

[23] Villoz F., Filippini T., Ortega N., Kopp-Heim D., Voortman T., Blum M.R., Del Giovane C., Vinceti M., Rodondi N., Chocano-Bedoya P.O. (2024). Dairy Intake and Risk of Cognitive Decline and Dementia: A Systematic Review and Dose-Response Meta-Analysis of Prospective Studies. Adv. Nutr..

[24] Mazzoleni E., Vinceti M., Costanzini S., Garuti C., Adani G., Vinceti G., Zamboni G., Tondelli M., Galli C., Salemme S. (2023). Outdoor artificial light at night and risk of early-onset dementia: A case-control study in the Modena population, Northern Italy. Heliyon.

[25] Jarmolowicz A.I., Chen H.Y., Panegyres P.K. (2015). The patterns of inheritance in early-onset dementia: Alzheimer’s disease and frontotemporal dementia. Am. J. Alzheimers Dis. Other Demen.

[26] Filippini T., Vinceti M. (2023). Social disparities and unhealthy lifestyles increase risk of dementia, particularly at a young age. Lancet Healthy Longev..

[27] Kauko A., Engler D., Niiranen T., Ortega-Alonso A., Schnabel R.B. (2024). Increased risk of dementia differs across cardiovascular diseases and types of dementia—Data from a nationwide study. J. Intern. Med..

[28] Wolters F.J., Segufa R.A., Darweesh S.K.L., Bos D., Ikram M.A., Sabayan B., Hofman A., Sedaghat S. (2018). Coronary heart disease, heart failure, and the risk of dementia: A systematic review and meta-analysis. Alzheimers Dement..

[29] Deckers K., Schievink S.H.J., Rodriquez M.M.F., van Oostenbrugge R.J., van Boxtel M.P.J., Verhey F.R.J., Kohler S. (2017). Coronary heart disease and risk for cognitive impairment or dementia: Systematic review and meta-analysis. PLoS ONE.

[30] Song R., Pan K.Y., Xu H., Qi X., Buchman A.S., Bennett D.A., Xu W. (2021). Association of cardiovascular risk burden with risk of dementia and brain pathologies: A population-based cohort study. Alzheimers Dement..

[31] Kogelschatz B., Zenger B., Steinberg B.A., Ranjan R., Jared Bunch T. (2024). Atrial fibrillation and the risk of early-onset dementia and cognitive decline: An updated review. Trends Cardiovasc. Med..

[32] Giannone M.E., Filippini T., Whelton P.K., Chiari A., Vitolo M., Boriani G., Vinceti M. (2022). Atrial Fibrillation and the Risk of Early-Onset Dementia: A Systematic Review and Meta-Analysis. J. Am. Heart Assoc..

[33] Zhang W., Liang J., Li C., Gao D., Ma Q., Pan Y., Wang Y., Xie W., Zheng F. (2023). Age at Diagnosis of Atrial Fibrillation and Incident Dementia. JAMA Netw. Open.

[34] Mazzola P., Rimoldi S.M., Rossi P., Noale M., Rea F., Facchini C., Maggi S., Corrao G., Annoni G. (2016). Aging in Italy: The Need for New Welfare Strategies in an Old Country. Gerontologist.

[35] Cortesi P.A., Fornari C., Madotto F., Conti S., Naghavi M., Bikbov B., Briant P.S., Caso V., Crotti G., Johnson C. (2021). Trends in cardiovascular diseases burden and vascular risk factors in Italy: The Global Burden of Disease study 1990–2017. Eur. J. Prev. Cardiol..

[36] Filippini T., Adani G., Malavolti M., Garuti C., Cilloni S., Vinceti G., Zamboni G., Tondelli M., Galli C., Costa M. (2020). Dietary habits and risk of early-onset dementia in an Italian case-control study. Nutrients.

[37] American Psychiatric Association (2013). Diagnostic and Statistical Manual of Mental Disorders: DSM-5™.

[38] Gorno-Tempini M.L., Hillis A.E., Weintraub S., Kertesz A., Mendez M., Cappa S.F., Ogar J.M., Rohrer J.D., Black S., Boeve B.F. (2011). Classification of primary progressive aphasia and its variants. Neurology.

[39] Rascovsky K., Hodges J.R., Knopman D., Mendez M.F., Kramer J.H., Neuhaus J., van Swieten J.C., Seelaar H., Dopper E.G., Onyike C.U. (2011). Sensitivity of revised diagnostic criteria for the behavioural variant of frontotemporal dementia. Brain.

[40] Filippini T., Tesauro M., Fiore M., Malagoli C., Consonni M., Violi F., Iacuzio L., Arcolin E., Oliveri Conti G., Cristaldi A. (2020). Environmental and Occupational Risk Factors of Amyotrophic Lateral Sclerosis: A Population-Based Case-Control Study. Int. J. Environ. Res. Public Health.

[41] Escudero-Martinez I., Morales-Caba L., Segura T. (2023). Atrial fibrillation and stroke: A review and new insights. Trends Cardiovasc. Med..

[42] Choi S.E., Sagris D., Hill A., Lip G.Y.H., Abdul-Rahim A.H. (2023). Atrial fibrillation and stroke. Expert. Rev. Cardiovasc. Ther..

[43] Buhari H., Fang J., Han L., Austin P.C., Dorian P., Jackevicius C.A., Yu A.Y.X., Kapral M.K., Singh S.M., Tu K. (2024). Stroke risk in women with atrial fibrillation. Eur. Heart J..

[44] Ferencz B., Gerritsen L. (2015). Genetics and underlying pathology of dementia. Neuropsychol. Rev..

[45] Nordstrom P., Nordstrom A., Eriksson M., Wahlund L.O., Gustafson Y. (2013). Risk factors in late adolescence for young-onset dementia in men: A nationwide cohort study. JAMA Intern. Med..

[46] Kuzma E., Lourida I., Moore S.F., Levine D.A., Ukoumunne O.C., Llewellyn D.J. (2018). Stroke and dementia risk: A systematic review and meta-analysis. Alzheimers Dement..

[47] van Gennip A.C.E., van Sloten T.T., Fayosse A., Sabia S., Singh-Manoux A. (2024). Age at cardiovascular disease onset, dementia risk, and the role of lifestyle factors. Alzheimers Dement..

[48] Cannistraro R.J., Badi M., Eidelman B.H., Dickson D.W., Middlebrooks E.H., Meschia J.F. (2019). CNS small vessel disease: A clinical review. Neurology.

[49] Lyon M., Fullerton J.L., Kennedy S., Work L.M. (2024). Hypertension & dementia: Pathophysiology & potential utility of antihypertensives in reducing disease burden. Pharmacol. Ther..

[50] Li G., Rhew I.C., Shofer J.B., Kukull W.A., Breitner J.C., Peskind E., Bowen J.D., McCormick W., Teri L., Crane P.K. (2007). Age-varying association between blood pressure and risk of dementia in those aged 65 and older: A community-based prospective cohort study. J. Am. Geriatr. Soc..

[51] Goulay R., Mena Romo L., Hol E.M., Dijkhuizen R.M. (2020). From Stroke to Dementia: A Comprehensive Review Exposing Tight Interactions Between Stroke and Amyloid-beta Formation. Transl. Stroke Res..

[52] Lopes M.A., Firbank M.J., Widdrington M., Blamire A.M., Kalaria R.N., O’Brien J.T. (2012). Post-stroke dementia: The contribution of thalamus and basal ganglia changes. Int. Psychogeriatr..

[53] Kalaria R.N., Akinyemi R., Ihara M. (2016). Stroke injury, cognitive impairment and vascular dementia. Biochim. Biophys. Acta.

[54] Pinho J., Quintas-Neves M., Dogan I., Reetz K., Reich A., Costa A.S. (2021). Incident stroke in patients with Alzheimer’s disease: Systematic review and meta-analysis. Sci. Rep..

[55] Rossor M.N., Fox N.C., Mummery C.J., Schott J.M., Warren J.D. (2010). The diagnosis of young-onset dementia. Lancet Neurol..

[56] Hu J., Fang M., Pike J.R., Lutsey P.L., Sharrett A.R., Wagenknecht L.E., Hughes T.M., Seegmiller J.C., Gottesman R.F., Mosley T.H. (2023). Prediabetes, intervening diabetes and subsequent risk of dementia: The Atherosclerosis Risk in Communities (ARIC) study. Diabetologia.

[57] Zhang J., Chen C., Hua S., Liao H., Wang M., Xiong Y., Cao F. (2017). An updated meta-analysis of cohort studies: Diabetes and risk of Alzheimer’s disease. Diabetes Res. Clin. Pract..

[58] Randväli M., Toomsoo T., Šteinmiller J. (2024). The Main Risk Factors in Type 2 Diabetes for Cognitive Dysfunction, Depression, and Psychosocial Problems: A Systematic Review. Diabetology.

[59] Titcomb T.J., Richey P., Casanova R., Phillips L.S., Liu S., Karanth S.D., Saquib N., Nuno T., Manson J.E., Shadyab A.H. (2024). Association of type 2 diabetes mellitus with dementia-related and non-dementia-related mortality among postmenopausal women: A secondary competing risks analysis of the women’s health initiative. Alzheimers Dement..

[60] Aldrugh S., Sardana M., Henninger N., Saczynski J.S., McManus D.D. (2017). Atrial fibrillation, cognition and dementia: A review. J. Cardiovasc. Electrophysiol..

[61] Kim D., Yang P.S., Lip G.Y.H., Joung B. (2020). Atrial fibrillation Increases the risk of early-onset dementia in the general population: Data from a population-based cohort. J. Clin. Med..

[62] Rusanen M., Kivipelto M., Levalahti E., Laatikainen T., Tuomilehto J., Soininen H., Ngandu T. (2014). Heart diseases and long-term risk of dementia and Alzheimer’s disease: A population-based CAIDE study. J. Alzheimers Dis..

[63] An J., Bider Z., Luong T.Q., Cheetham T.C., Lang D.T., Fischer H., Reynolds K. (2021). Long-Term Medication Adherence Trajectories to Direct Oral Anticoagulants and Clinical Outcomes in Patients With Atrial Fibrillation. J. Am. Heart Assoc..

[64] Cadogan S.L., Powell E., Wing K., Wong A.Y., Smeeth L., Warren-Gash C. (2021). Anticoagulant prescribing for atrial fibrillation and risk of incident dementia. Heart.

[65] Bunch T.J. (2020). Atrial Fibrillation and Dementia. Circulation.

[66] Zamboni G., Maramotti R., Salemme S., Tondelli M., Adani G., Vinceti G., Carbone C., Filippini T., Vinceti M., Pagnoni G. (2024). Age-specific prevalence of the different clinical presentations of AD and FTD in young-onset dementia. J. Neurol..

