# Supplementary material for: Atrial Fibrillation and Other Cardiovascular Factors and the Risk of Dementia: An Italian Case–Control Study
Source: Int J Environ Res Public Health. 2024 May 27;21(6):688. doi: 10.3390/ijerph21060688 (PMC11203794; doi:10.3390/ijerph21060688)
Supplement: Supplementary file 1 [file ijerph-21-00688-s001.zip › ijerph-3027283-Supplementary Materials.pdf]

International Journal of
*Environmental Research
and Public Health*

MDPI

SUPPLEMENTAL MATERIAL

Table S1. Clinical diagnosis of early-onset (EOD) and late-onset (LOD) dementia cases.

**Diagnosis**	**EOD N (%)**	**LOD N (%)**
Total cases	58 (100)	34 (100)
Alzheimer's dementia	32 (55.2)	25 (73.5)
Frontotemporal dementia	19 (32.8)	2 (5.9)
Frontotemporal dementia	17 (29.3)	2 (5.9)
Progressive Supranuclear Palsy	2 (3.4)	-
Vascular dementia	5 (8.6)	1 (2.9)
Cerebral Amyloid Angiopathy	1 (1.7)	1 (2.9)
Lewy's body dementia	1 (1.7)	3 (8.82)
Parkinson disease with dementia	-	1 (2.9)
Normal-pressure hydrocephalus	-	1 (2.9)
